# Law Cases Bearing upon the Subject of Insanity

**Published:** 1856-03

**Authors:** 


### *Law Cases Bearing upon the Subject of Insanity.*

Translated from the French.

The following is extracted from the American Journal of Insanity:

*Homicide.—Hallucinations of Hearing.*—The Court of Assizes of Pas-de-Calais has recently condemned to twenty years' hard labor, a monomaniac, who, under the influence of hallucinations of hearing, had been guilty of several attempts at homicide, and had finally committed a murder, without either motive or interest. The accused is an Englishman, named Piers, aged forty-four, and for twenty-five years a resident of St. Omer. His character had been irreproachable: he was kind and amiable, but always exceedingly sensitive. The witnesses spoke of him as a strange man, who allowed himself to become angry under the most trifling circumstances. He had long suffered from hallucinations. He often charged those around him with expressions they had never used; and one day, three men happening to meet and converse beneath his window, he at once fancied they were speaking prejudicially of himself, and fired two pistol shots at them, fortunately without effect. These men, at the trial, testified that they had not referred to him at all.

On the 17th of April last, the proprietor of the house in which he lived was talking in the yard with one of his neighbors, a merchant, like himself. They spoke of their own business, and were not even thinking of the unhappy man, when the latter, who was shut up in his room, where he could not hear, and could only see them through the glass in the window, fancied that they were speaking of him, and that they had grossly insulted him. He thought he heard his landlord distinctly say, "He is without his pantaloons; he is a *bougre.*" At once resolving to be revenged, he had, like the greater portion of the insane, recourse to a ruse, and opening the window, politely asked him to come in. The latter, without suspicion, accepted the invitation, but had scarcely entered, when Piers spoke to him passionately, and demanded the grounds of the slander he had just uttered against him. While the unfortunate landlord was protesting against this charge, and denying that he had spoken a single word to his prejudice, Piers seized a pistol, and wounded him mortally.

The neighbors, collecting together at the sound of fire-arms, were at first repulsed by the murderer, who kept them at bay with a gun, and forbade them to cross the threshold of his door; but a courageous citizen finally rushed upon and disarmed him. The murder was commited with perfect composure: this indifference did not forsake the accused for an instant; and he related before the Commissary of Police each circumstance as it had occurred, boasting of his deed, and evincing no regret at its commission. The same unconcern marked his conduct during the trial. He declared that he had heard distinctly the insult that was addressed to him, and that it would have been a dishonor to himself if he had not avenged it. No other excuse was offered, and he several times repeated that he had only done his duty in committing the murder.

"When you induced the unfortunate Berthier to enter your room," asked the President, "what was your intention?" "I had firmly resolved to kill him," replied Piers. "The act that you have committed is considered murder in all countries: is it not the greatest of crimes?" "The reflection," said he, "that was cast upon me was infinitely more serious than my deed: it was the grossest outrage that could be inflicted upon a man, and no one, without being dishonored, could allow him to live who had offered it." "Were you in similar circumstances, would you again act in the same way?" The accused unhesitatingly, and with assurance replied, "Yes, sir."

The jury, as we have said, convicted him, but *under extenuating circumstances.* Three physicians, appointed to examine the prisoner, both before and during the trial, declared unanimously that this unhappy man was the puppet of his hallucinations—that these errors in hearing had led him to the commission of a criminal act, the consequences of which he evidently did not appreciate; and, finally, that he should be placed in an asylum for the insane, and not be sent either to the scaffold or the galleys. But this failed to convince either the prosecuting attorney or the jurors, and the galleys will possess one monomaniac the more.

We have, in this Journal, so often treated the subject of homicidal monomania, that it would be superfluous to dwell longer on this case, which is, however, in more than one respect remarkable. The hallucinations of the accused were not doubted at the trial; testimony was too abundant; but the court was unwilling to allow that, at the moment of the commission of the crime, he had not free liberty of action. They attached particular importance to the artifice used to entice his unfortunate victim into his chamber. He declared that if he did not fire from the window to kill his victim immediately, it was only because he was afraid of missing him; and the jury could not believe that a man capable of such reasoning was a lunatic, because it is generally believed that lunatics do not reason. We are acquainted with the sad results of this erroneous belief.

Artifice and dissimulation are, on the contrary, characteristic of monomania; and it sometimes requires great skill to fathom the intentions of an insane person. Professional men alone can recognize these; and unfortunately their advice is not always followed, even by those who consult them. We ought, however, to say, that magistrates have for some years past studied these important and delicate points, and the number of insane indicted is infinitely smaller than formerly. It is left for our *confrères*, consulted in these obscure cases, to complete their conviction, as much by their reserve, when they are not themselves sufficiently enlightened, as by the clearness of their conclusions when the accused do not seem responsible for their acts. Time and observation will hasten the reform already commenced, and soon our eyes will be no longer saddened by the sight of wretched lunatics smitten down by justice, and the objects of persecutions they cannot comprehend.—*Journal de Médecine,* July, 1855.

*Homicidal Propensity developed during Pregnancy.*—A criminal case, presenting a question of great interest in legal medicine, has recently been brought to trial at the Court of Assizes at Aube. A young woman, of irreproachable character, tenderly attached to her husband, and living with him upon the best of terms, was, nevertheless, accused of attempting to poison him. This unhappy woman denied none of the acts with which she was charged by the prosecution; but her counsel held that the state of pregnancy in which she had been for a short time, had perverted her moral faculties, and not left her the free arbiter of her actions. We have here an extract from the indictment, which the depositions of the witnesses and the confessions of the accused fully confirmed.

On the 30th of October, 1854, a man named Baudry, recently discharged from military service, married Louise Yvan, aged eighteen.— They resided in the hamlet of Dival, a commune of Villenauxe; and everything, to the eyes of the relatives of the married couple, who frequently visited them, gave proof that they were living together on the best of terms. Sometimes, however, Baudry was struck by strange expressions he heard from his wife. Towards the close of December, while both were busy at their work, she said to her husband, without anything giving occasion for such a remark, "You will die this year; I shall die also."

On the 3d of January, Baudry, after finishing his work, came in to supper about six o'clock in the evening. After his meal, he went to the cupboard for the remainder of some prunes cooked the previous evening, and of which he had eaten the same day at dinner with his mother and his wife. He ate five or six, in which he perceived a bitter taste, which surprised him. The last he put into his mouth caused much pain in his tongue, and he said to his wife, "These prunes are spoiled or poisoned." "Who," she replied, "do you think has poisoned them?" Upon his suggesting that the disagreeable taste, without doubt, proceeded from leaving the prunes in the same dish with the juice, she eagerly availed herself of the explanation, and said, "That is probable." She then put a prune to her own lips, but soon rejected it, saying she should vomit. "You see," said Baudry, "that these prunes are poisoned;" but she did not reply. He proposed keeping them for her niece to eat the following day; but she said it was not necessary to expose that child to sickness, also, and hurried to throw them out of doors. An hour after his repast, Baudry suffered from a violent headache, accompanied by a burning sensation in the stomach, and throughout the night experienced all the symptoms consequent upon the administration of poison. During the whole of this time his wife slept, or pretended to sleep, in the same bed.

The next morning, at six o'clock, Baudry, in arising, placed his hand, in the darkness, upon the pocket of his wife's dress, and felt that it contained little packages rolled up in paper. These inclosed pieces of blue vitriol, and smoking tobacco mixed with powdered vitriol. He said to his wife, "You wish, then, to poison me?" "Yes," she replied, "I have had an idea of the kind. I'm guilty—kill me."

Prompt remedies soon placed Baudry out of danger. In the presence of the Commissary of Police, and before the neighbors, his wife renewed her avowals. She acknowledged that, having concluded to kill her husband, she had bought some vitriol at the grocer's on the 29th of December. Questioned as to the motive which had induced her to commit the crime, she replied: "For three weeks my husband found fault with all I did: nothing was right. I was full of spite. I regretted the loss of my liberty, and wished to recover it by putting my husband to death." She finally said that she was pregnant, and it was, perhaps, that which had inspired her with the idea of poisoning him.

This is the manner in which she related the facts before the court: "I cannot tell whence I got the idea. We kept house pleasantly together. It was on Thursday, December 28th, that this thought took possession of me—how, I cannot explain. I was unable to resist it, and formed my resolution. The next day, on my way to market at Villenauxe, I asked the grocer, Relif, for a sous-worth of vitriol, and he gave me five or six pieces, each of the size of a large hazel nut. I put them into the pocket of my skirt, where I kept them until the moment of using them. On Thursday evening, January 2d, after scraping one of the pieces of vitriol, I put it in my husband's smoking tobacco, which he had forgotten, in the cupboard, and the remainder in the prunes, which were to be served next day."

This woman was of Russian origin. Her father, who remained in France after the invasion of 1814, had the reputation of being cruel towards animals, and the accused herself was of a gloomy disposition, and a little harsh. She was industrious, of little intelligence, and sometimes appeared idiotic to such a degree that the children ran after her in the streets. Her conduct had in other respects, been invariably good, and her husband attributing, as did also their neighbors, her criminal action to her pregnancy, insisted that she should be restored to him.

The *ministere public* sustained the prosecution, by questioning how far her pregnancy had controlled the freedom of action of this woman; but the jury, after a few moments' deliberation, brought in a verdict of acquittal.—*Journal de Medecine*, May, 1855.

*Homicide —Mental Alienation.*—The *Gazette Medicale de Lyon* publishes the following account of Jeanne Desroches, who acquired at the time a sad judicial celebrity, and afterwards died at the Asylum of Antiquaille, after a residence of more than twenty years.

On Tuesday, June 2d, 1832, Jeanne Desroches, who had been married eight years, went from her own dwelling to the village where her mother lived. On the way she entered the house of a couple named Champart, where there were two very young children. She killed one with a knife: the child uttered a single cry and died. After this murder she ran to her mother's house, found her in the stable, gave her a violent blow with a knife, threw her down, and killed her with a pickaxe. She entered a neighboring house, the widow George's, and struck her also several times with the knife. She afterwards went to the house of a woman named Dorneron, and diverting her attention, darted upon her child, inflicting upon its neck a large wound, which was followed by a fatal hemorrhage. She also tried to murder the woman Dorneron, but her resistanee was too vigorous. Seeing that she was not able to throw her down, she fled to her mother's house, went into the cellar, drew the bung from a cask, and threw into it the instrument of so many murders. She was arrested a few minutes afterwards, and brought before the assizes of the Rhone. Notwithstanding the deposition of Dr. Bottex, this unhappy woman, who had previously given unequivocal signs of mental disease, was declared guilty of paricide and three premeditated homicides, under extenuating circumstances, and condemned to ten years' hard labor. Soon after her commitment, in a paroxysm of fury, she tore off the ends of two of her fingers with her teeth. After passing about six months in the central house at Montpelier, and nine years and a half in the asylum for the insane of this city, she was transferred to the Antiqua lle. From 1842 to 1852, her lucid intervals were more frequent, and one day this unfortunate woman related to her physician, with poignant emotion, even to the minutest details, the events of that frightful morning, during which she killed, among other persons, her mother, "whom she most loved, after her God." Like all the insane, she regretted, but without repentance, since she had acted "in a moment of forgetfulness." She was in all other respects a very honest and highly esteemed woman. She was of robust constitution; was subject to epistaxis; and had had a slight attack a few hours before the murder.

From 1852 until her death she became gradually worse. The lucid intervals were more rare, and the maniacal excitement more persistent. During the year 1854, there was, so to speak, no intermission. She changed a little at the approach of death, as is frequently the case with the insane. This maniacal excitement, with general delirium, incoherence of ideas, &c., was remarkable in this respect—that, under the influence of the least contradiction, or even without any apparent, appreciable cause, she took the character of a true, furious maniac, and her physiognomy assumed a singular expression of ferocity. Nevertheless, no act and no attempt has ever been witnessed to recall the circumstances which marked the access of the disease.—*Journal de Medecine*, May, 1855.

